# Preparation and evaluation of inhalable itraconazole chitosan based polymeric micelles

**DOI:** 10.1186/2008-2231-20-85

**Published:** 2012-12-03

**Authors:** Esmaeil Moazeni, Kambiz Gilani, Abdolhossein Rouholamini Najafabadi, Mohamad reza Rouini, Nasir Mohajel, Mohsen Amini, Mohammad Ali Barghi

**Affiliations:** 1 Aerosol Research Laboratory, Department of Pharmaceutics, School of Pharmacy, Tehran University of Medical Sciences, Tehran, Iran; 2 Department of Pharmaceutics, School of Pharmacy, Tehran University of Medical Sciences, Tehran, Iran; 3 Department of Medicinal Chemistry, School of Pharmacy and Drug Design & Development Research Center, Tehran University of Medical Sciences, Tehran, Iran; 4 XRD Research Laboratory, School of Sciences, Tehran University, Tehran, Iran

**Keywords:** Polymeric micelles, Itraconazole, Chitosan, Nebulization, Pulmonary drug delivery

## Abstract

**Background:**

This study evaluated the potential of chitosan based polymeric micelles as a nanocarrier system for pulmonary delivery of itraconazole (ITRA).

**Methods:**

Hydrophobically modified chitosan were synthesized by conjugation of stearic acid to the hydrophilic depolymerized chitosan. FTIR and ^1^HNMR were used to prove the chemical structure and physical properties of the depolymerized and the stearic acid grafted chitosan. ITRA was entrapped into the micelles and physicochemical properties of the micelles were investigated. Fluorescence spectroscopy, dynamic laser light scattering and transmission electron microscopy were used to characterize the physicochemical properties of the prepared micelles. The in vitro pulmonary profile of polymeric micelles was studied by an air-jet nebulizer connected to a twin stage impinger.

**Results:**

The polymeric micelles prepared in this study could entrap up to 43.2±2.27 μg of ITRA per milliliter. All micelles showed mean diameter between 120–200 nm. The critical micelle concentration of the stearic acid grafted chitosan was found to be 1.58×10^-2^ mg/ml. The nebulization efficiency was up to 89% and the fine particle fraction (FPF) varied from 38% to 47%. The micelles had enough stability to remain encapsulation of the drug during nebulization process.

**Conclusions:**

In vitro data showed that stearic acid grafted chitosan based polymeric micelles has a potential to be used as nanocarriers for delivery of itraconazole through inhalation.

## Background

Invasive pulmonary fungal infection is major infectious disease among immunosuppressed patients. Difficulty of early clinical diagnosis and poor response to the common antifungal treatment result in high mortality percent of patients. In the recent years many efforts done to found an alternative drug delivery strategies for antifungal agents [1,2]. Aerosolization of antifungal agents can be used for prophylaxis against pulmonary fungal infections. High drug concentration in the site of infection, non invasive route of administration and reduce systemic toxicities are some advantages of this route of administration [3].

While amphotericin is an effective drug for treatment of fungal infections, its adverse effects reduces its potential uses and particular interest has been focused on the use of other antifungal drugs [4].

Itraconazole (ITRA), a triazole member, is a highly hydrophobic drug (water solublility ~1 ng/ml at neutral pH and 4 μg/ml at pH 1) [5] which has broad spectrum of activity against a number of fungal species [6].

Most of potent antifungal compounds like amphotericin and azoles have very low solubility. Approaches for achieving complete dissolution of ITRA often have disadvantages associated with large quantities of required excipients [7]. These strategies may cause some limitation due to the toxicity induced by high concentrations of the drug or excipients like 2-hydroxypropyl-β-cyclodexterin. Scientists have been used different strategies to obtain technologies which required lower amounts of excipients to increase the solubility. Various solid dispersion, emulsification and nanotechnology approaches have been widely used to increase the solubility of itraconazole. Akkar and Muller prepared intravenous itraconazole emulsion based on solEmuls technology. Using this technology, the drug localizes in the interfacial lecithin layer of the emulsions by homogenising a hybrid dispersion of oil droplets and drug nanocrystals in water [8]. Also Keck and Muller used modified high pressure hemogenisation technique (NANOEDGE) to prepare nanocrystals of poorly water soluble drugs like itraconazole [9]. Some studies based on evaporative precipitation into aqueous solution (EPAS) or spray freezing into liquid (SFL) techniques has been used to obtain nano itraconazole for pulmonary delivery systems [10-12]. McConville and co-workers nebulized itraconazole dispersion prepared by EPAS or SFL techniques and obtained high lung concentration in animal study [11].

Self-assembled polymeric micelles (PM) are widely investigated as an alternative choice in the development of delivery systems for poorly water soluble drugs. PMs are composed of different amphiphilic copolymers. The micelles prepared by these copolymers in the aqueous medium contain internal hydrophobic segments as drug reservoir and external hydrophilic segments as surrounding shell [13]. PMs based on chitosan, a natural biodegradable cationic polysaccharide [14], with various modification by different hydrophobic groups have been used for encapsulation of different hydrophobic drugs. Doxorubicin [15], camptothecin [16], all-trans retinoic acid [17] and paclitaxel [18] are some hydrophobic drugs that encapsulated by chitosan based polymeric micelles. There are only few reports investigating the application of inhalable polymeric micelles for delivery of all trans retinoic acid, amphotericin B and budesunide [19-21].

The ability of the modified chitosan polymeric micelles to improve ITRA solubility and pulmonary delivery of ITRA were investigated in this research. In current study, depolymerized chitosan (Mw 24 kDa) was chemically modified by stearic acid and its properties were characterized by ^1^HNMR and FT-IR. In vitro nebulization parameters like fine particle fraction (FPF), nebulization efficiency (NE) and drug remained encapsulated in the micelles (DRE) after nebulization process were studied.

## Methods

### Materials

Itraconazole was kindly supplied by Hetero, India. Chitosan (Medium molecular weight, 95% deacetylated) was obtained from Primex, Iceland. Stearic acid, KCl, NaOH, glacial acetic acid, sodium nitrite_,_ 1-ethyl-3-(3-dimethylaminopropyl) carbodiimide (EDC), diethylene amin (DEA), pyrene and all other solvents in analytical and HPLC grade were provided from Merck, Germany.

### Depolymerization of chitosan

Chitosan was chemically depolymerized by sodium nitrite at room temperature according to Mao et al. pervious report [22]. Medium molecular weight chitosan was dissolved in 1% acetic acid and then appropriate amount of 0.1 M NaNO_2_ was added at room temperature. The reaction was performed for 3 hrs under nitrogen gas. To precipitate depolymerized chitosan, the pH value of reaction was increased to about 8 with NaOH. The obtained chitosan after centrifugation was dialyzed (MWCO 8 kDa, Spectrum Laboratories, USA) against deionized water for 48 hrs and finally lyophilized (Christ, α2-4, Germany) at −80°C and 0.001 mbar pressure for 48 hrs. The molecular weight of obtained chitosan was determined by size exclusion chromatography using Knauer apparatus (Berlin, Germany) utilized with a PL Aquagel-OH Mixed column (25 mm ID 8 μm at 25°C) and a flow rate of 4 ml/min in acetate buffer medium (0.2 M acetic acid and 0.1 M sodium acetate with pH value of 4.6 ± 0.05) as diluent.

### Synthesis and characterization of stearic acid grafted to depolymerized chitosan

Stearic acid grafted to depolymerized chitosan was synthesized as previously reported method [18]. Briefly, 30 ml aqueous solution of the depolymerized chitosan was prepared at concentration of 30 mg/ml. Stearic acid (50 mg/ml) and EDC (3 mol/mol stearic acid) were dissolved in absolute ethanol and the mixture was added to the chitosan solution under stirring at 80°C for 5 hrs and the reaction was stirred further 24 hrs at room temperature. The mixture then was dialyzed (8 kDa dialysis tube, Spectrum laboratories, USA) against distilled water containing 10% v/v ethanol for 48 hrs and finally the dialysis was continued against distilled water for 2 hrs. The final product was lyophilized (Christ, α2-4, Germany). FT-IR spectra were carried out using Nicolet (magna IR-550, USA) spectrometer by preparation of KBr disks. Bruker FT-500 ^1^H NMR (Bruker, Germany) spectra were used to characterize and calculate the substitution degree of modified chitosan.

### Critical micelle concentration (CMC) measurement

The fluorescence technique was used to measure the critical micelle concentration (CMC) of polymeric micelles, where pyrene was chosen as a fluorescence probe [23]. Pyrene (6×10^−7^ M) was added to each of a series of vials and then 10 ml of the modified chitosan solutions with various concentrations (250 ng/ml–2 mg/ml) were added. After 3 hrs stirring at room temperature, the fluorescence spectrum was obtained with a spectrofluorometer (Cecil 9000 series, England) and I_1_ and I_3_ were taken from the emission intensity of pyrene at 374 nm and 395 nm, respectively.

### Preparation and characterization of ITRA micellar formulations

Different ITRA micellar formulations were prepared by film hydration procedure [18,24]. Briefly, 10 mg or 20 mg of the modified chitosan and an appropriate amount of ITRA (5% to 50% weight of the polymer) were added to 100 ml of dichloromethane in a 250 ml round-bottomed flask. The mixtures were sonicated using a bath sonicator (Starsonic60, Liarre, Italy) to obtain nearly clear dispersions, then the solvents were removed using a rotary evaporator (Buchi Rotavapor R-124, Buchi, Switzerland) at a reduced pressure at 60°C and 120 rpm to obtain thin films. To remove residual amount of the solvents, the films were placed in a vacuum oven (Memmert, VO400, Germany) at 40°C overnight. The dried thin films were hydrated with 10 ml of deionized water (Direct-Q® 3UV, Millipore, France) for 45 min at 50°C and 120 rpm.

Finally, the suspensions were sonicated for 2 min by a probe sonicator (UP400S, hielscher ultrasound technology, Germany) in ice-bath.

To measure ITRA entrapment efficiency (EE), the final dispersions were filtered through 0.22 μm Durapore® filters (MILLEX®-GV, Millipore, Ireland) and specified amounts of the filtrates were added to 8 ml of DMF:water (80:20). Measurement of ITRA concentration was carried out using an HPLC system (Waters 600E, Millipore, USA) equipped with a C18 column (4.6 mm×150 mm, 5 μm, teknokroma, Spain). The mobile phase consisting of acetonitrile, water, and diethyl amine (60:40:0.05, v/v) was delivered at a flow rate of 1 ml/min, while UV detection was utilized at 260 nm. The injection volume was 20 μl. The drug EE was calculated as a percentage value by dividing the amount of ITRA entrapped in the micelles to the initial amount of ITRA. The drug loading (DL) value obtained as percent ratio of the weight of ITRA loaded in the micelles to the total weights of the micelles. The particle size distribution and the zeta potential of polymeric micelle formulations were measured by a Zetasizer (Nano-ZS, Malvern Instruments Ltd., UK).

### Nebulization efficacy of ITRA-loaded polymeric micelle formulations

Nebulization of the polymeric micelle formulations were done with an Air-jet nebulizer (Hudson®, UK). 5 ml of each formulation was placed into the nebulizer which was connected to the mouthpiece of a Twin stage impinger (TSI; Copley, UK) containing DMF/distilled water (3/1 vol.) in the stages 1 and 2. After connecting the TSI to a vacuum pump, the air flow (28.3 l/min) was adjusted through the apparatus. Nebulization was ended when no aerosol was produced. After nebulization, the two chambers of TSI, its mouthpiece and the nebulizer were washed separately with DMF/distilled water (3/1 vol.). The total amount of the ITRA recovered from the two stages of TSI was defined as the emitted dose. Nebulization efficiency (NE) was calculated as the percentage of the ratio of the emitted dose to the initial amount of the drug poured into the nebulizer. Fine particle fraction (FPF) was determined by dividing the amount of ITRA recovered from the lower stage of TSI (fine particle dose; FPD) to the emitted dose and expressed as percentage. Drug remained encapsulated in the micelles (DRE) at the end of the nebulization process was determined with minimum change in the procedure which was used for determination of the nebulization efficiency. Briefly, distilled water was located in the upper and lower chambers of TSI. After collection of the nebulized micelles, half of the dispersion was diluted four times with DMF to determine the total amount of the drug emitted from the nebulizer. The other half was filtered through a syringe filter (0.45 μm) to separate the non-encapsulated drug. Then, the filtrate was diluted four times with DMF. The percent of ITRA remained in the micelles during nebulization was calculated by dividing the concentration of ITRA in the filtrate to the total amount of the drug emitted from the nebulizer, expressed as percentage.

### In vitro release study

The in vitro release profile of ITRA from micelles were carried out by dialysis method [16].Briefly, 5 ml of each micellar formulation( 5% drug percent ratio with 1 and 2 mg/ml of polymer concentration) was placed in a dialysis tube (Mwco 8 kDa, Spectrum laboratories, USA) which was immersed in 20 ml of PBS (pH 7.4) in a bath shaker at 37°C under mild agitation. At predetermined time intervals, the whole medium was taken out and the same volume of fresh medium replaced. The amount of ITRA released from the micelles was determined by the HPLC method which has been described before.

### In vitro Anti fungal activity

ITRA minimal inhibitory concentration (MIC) was determined by broth macro dilution method in Sabouraud dextrose broth medium. The antifungal efficacy of two drug loaded polymeric micelle formulations prepared using 50% and 5% initial drug amount per mg of polymer weight at 2 mg/ml initial polymer concentration were tested against dissolved ITRA in DMSO. MIC was defined as the minimal concentration of the antifungal agent inhibiting visible growth of the microorganism. The concentration of ITRA used for MIC determination of each fungi varied from 4 to 0.03 μg/ml. In each tube containing 1 ml of broth medium and diluted ITRA formulation, 10^6^ CFU of fungal organisms (*Candida albicans, Aspergillus niger, Aspergillus fumigatus*) was added as an inoculum. The tubes with total volume of approximately 1 ml in each were incubated at 25°C for 72 h. The incubation time for *Candida albicans* was 48 h. All the tests were repeated three times.

### Statistical analysis

Data for all measurements were considered as mean ± standard deviation (S.D.) of three separate experiments. One-way analysis of variance (ANOVA) followed by Tukey post hoc test were used for statistical analysis of the results. The significance level was set at p < 0.05.

## Results and discussions

### Depolymerization of chitosan

**Figure 1:**
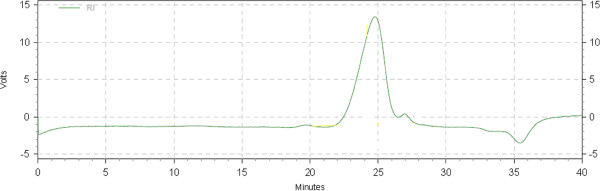
The GPC spectrum of depolymerized chitosan.

Depolymerization of chitosan by NaNO_2_ and H_2_O_2_ were previously investigated [22,25]. In this study, NaNO_2_ was used and the obtained depolymerized chitosan showed good water solubility. Figure 1 shown gel permeation chromatography (GPC) spectrum of depolymerized chitosan. The data of GPC revealed that the depolymerized chitosan had number average molecular weight (Mn) of 9560 K, weight average molecular weight (Mw) of 14984 K and Z-average molecular weight of 24378 K with a narrow molecular mass distribution with polydispersity index (Mw/Mn) of 1.56, indicating a monodispersed chain length.

### Synthesis and characterization of stearic acid grafted to depolymerized chitosan

The synthesis yield was about 41%. To confirm structural change and binding of stearic acid to the depolymerized chitosan, FT-IR and ^1^HNMR analysis were used.

**Figure 2:**
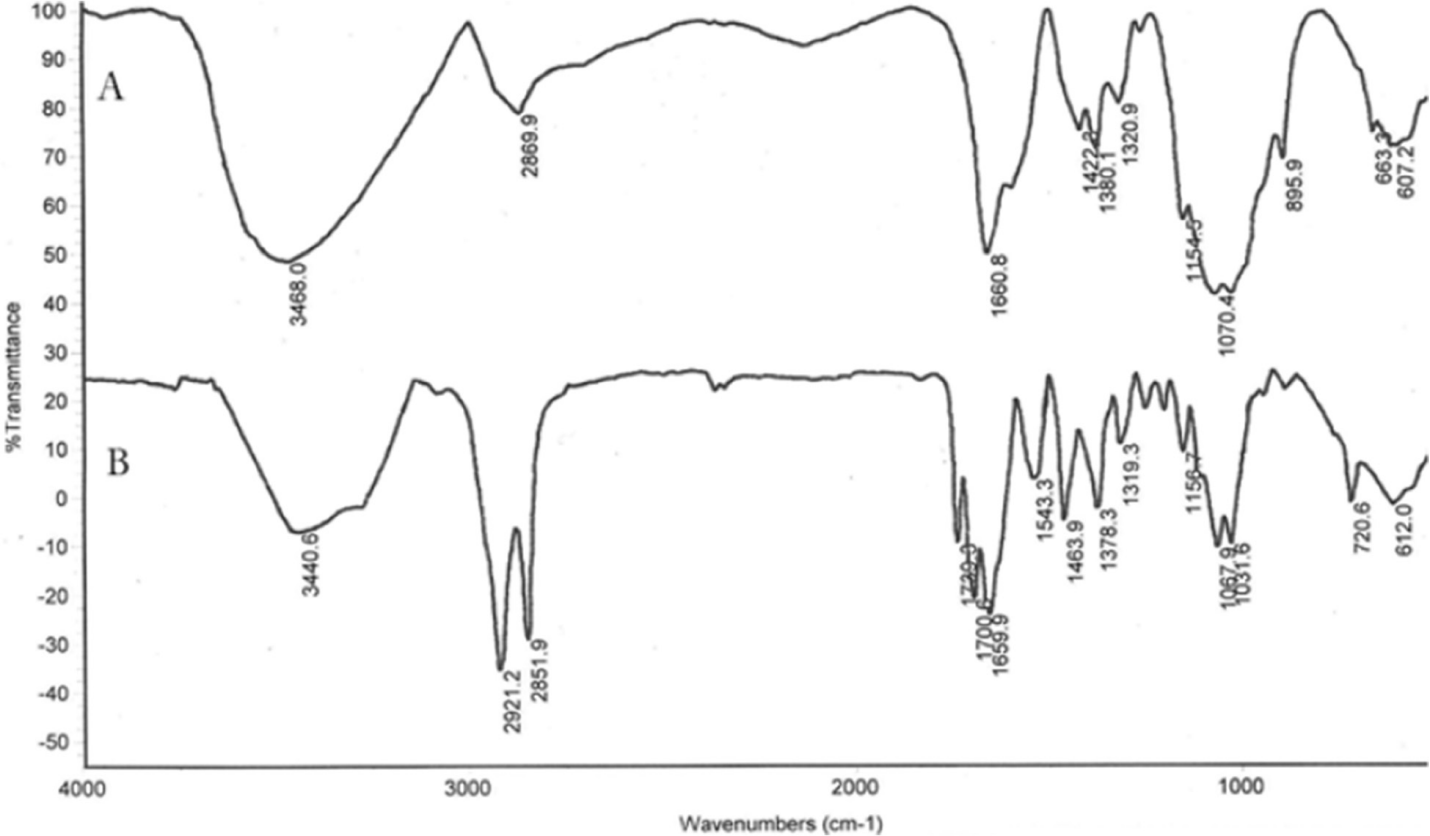
**FT**-**IR spectra of chitosan** (**A**) **and depolymeriezed chitosan**-**stearic acid** (**B**).

Compared to the IR spectrum of chitosan, the stearic acid-chitosan spectrum showed some changes according to the binding between stearic acid carboxylic group and chitosan amine (Figure 2). The increase of the C-H absorption at 2851.9 cm^-1^ indicated the presence of stearic acid. Stearic acid-chitosan showed two absorptions at 1700.6 cm^-1^ and 1739.3 cm^-1^ that ascribed to the carbonyl zone which related to the chemical and physical binding of stearic acid and chitosan. The purity of the synthesized polymers has been studied by using FT-IR spectrum of the samples, according to the similar studies. After dialysis of stearic acid grafted to the chitosan (against distilled water containing 10% v/v ethanol for 48 hrs) the free stearic acid removed from synthesized product. To confirm this result, the results obtained from washing synthesized product with warm ethanol on the 0.45 μm filter were compared and shown similar results as well as the dialysis method. Considering the low solubility of stearic acid in water, the trace of free stearic acid molecules remained in the aqueous media might find its way to the core hydrophobic segments of the micelles, the place that is thermodynamically more suitable for these molecules. But it is possible a very low percent of free stearic acid intercalate between natural polymer chain and cannot remove completely. The FT-IR spectrum after repeat of synthesis procedures were same and shown the suitability and repeatability of our procedure. All of these results indicated the formation of amide band and confirmed the conjugation of chitosan to stearic acid.

**Figure 3:**
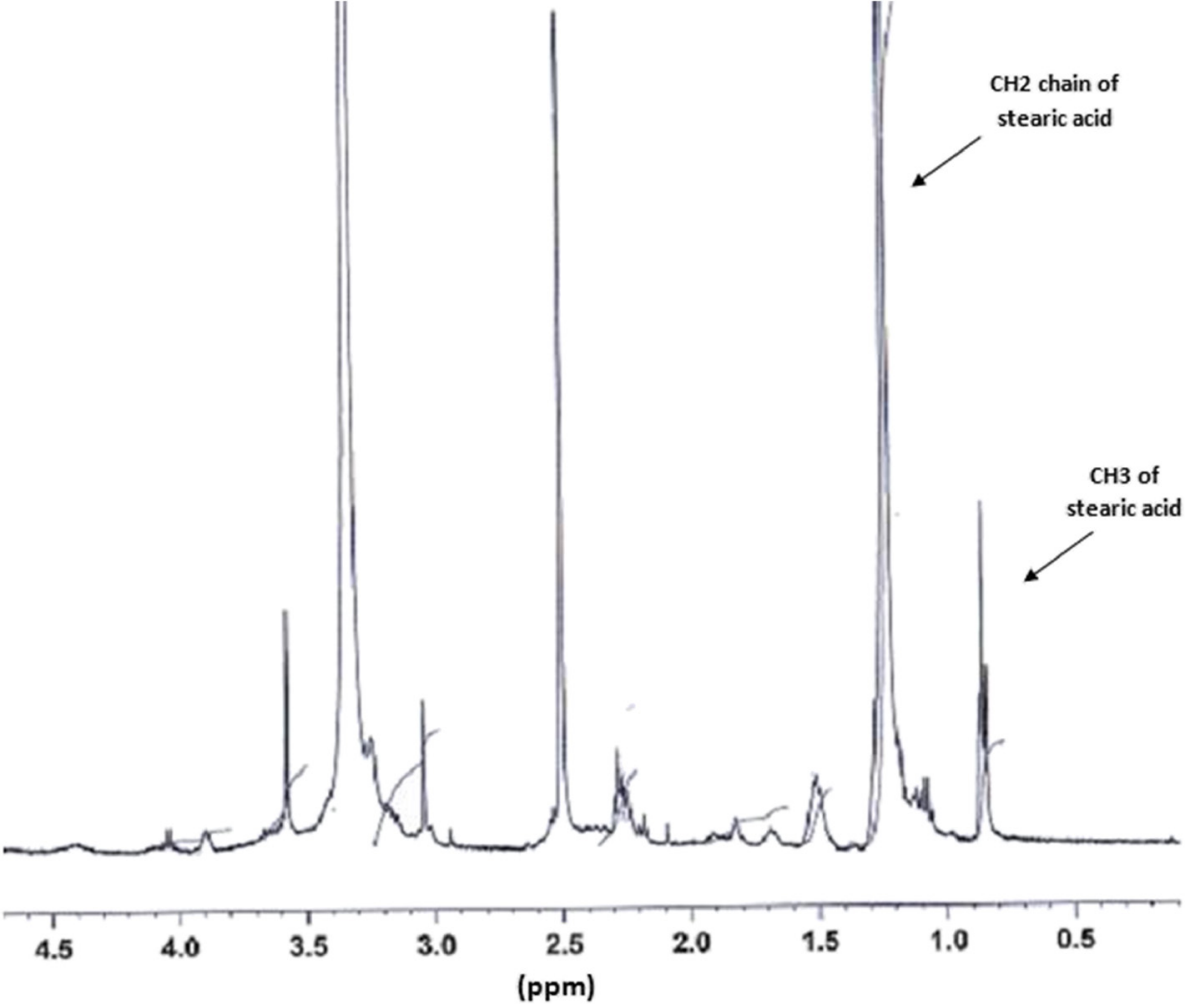
**HNMR spectra of depolymerized chitosan grafted to stearic acid in DMSO**-**d6.**

The ^1^H-NMR spectra of the depolymerized chitosan grafted to stearic acid showed in Figure 3. compared with chitosan ^1^H-NMR spectrum the triplet signals at 0.85 ppm, due to the terminal CH3 protons of stearic acid. The new peak at 1–1.5 ppm was corresponded to the CH2 chain of acyl chain. These results exhibited that stearic acid was linked to depolymerized chitosan.

The degree of substitution of the prepared modified polymer was about 61%, calculated by ^1^HNMR method. To calculate the degree of substitution, the area under the triplet signals at 0.85 ppm was compared to the area under the peaks at 2–4 ppm, which were related to hydrogens of the depolymerized chitosan.

### Critical micelle concentration (CMC) measurement

**Figure 4:**
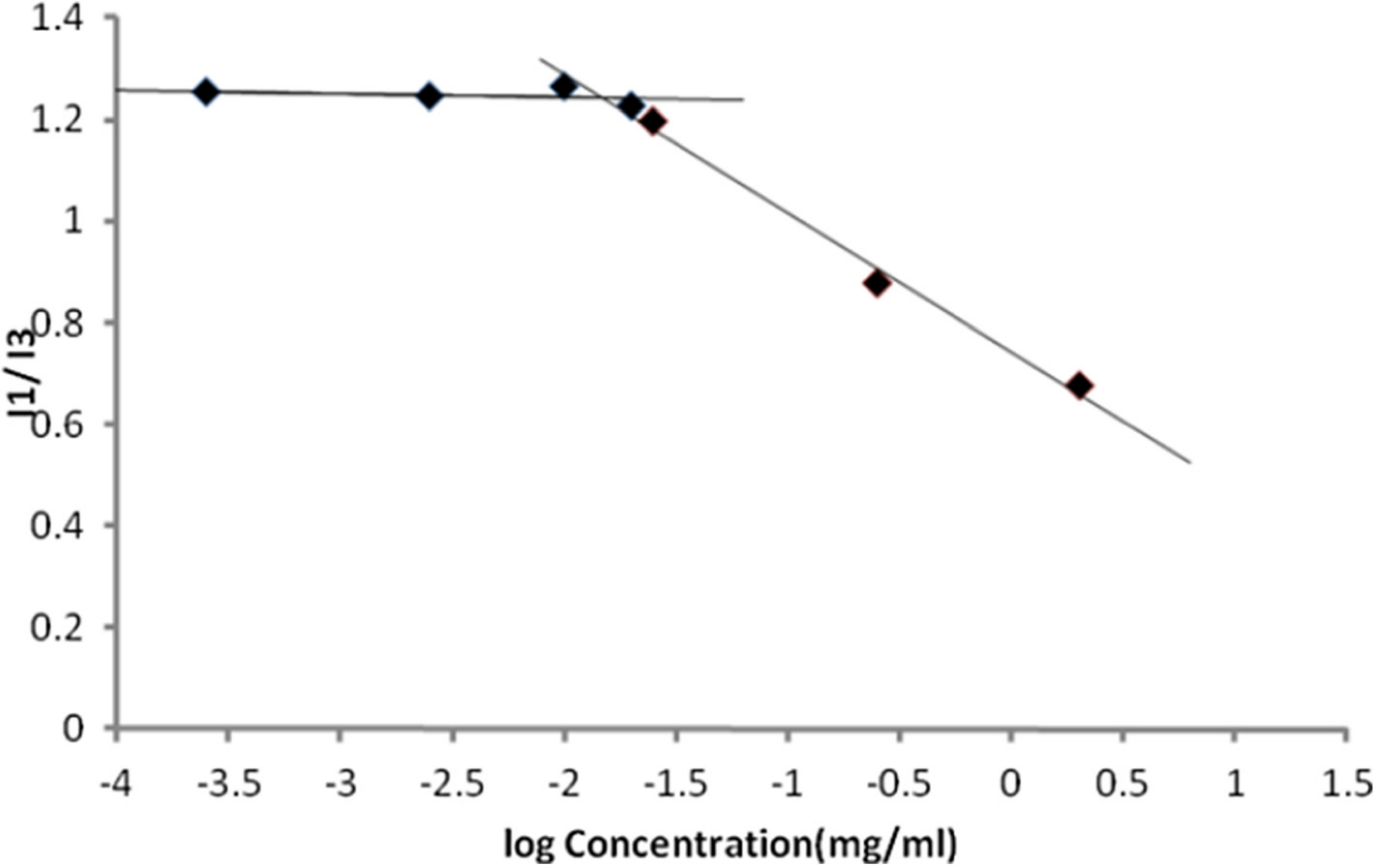
**Intensity ratio plot of 1374**/**1395 versus concentration of chitosan**-**stearic acid.**

Many researches exhibited that polymeric micelles generally have lower CMC than low molar weight surfactant micelles. When pyrene is in polar media, its emission is weak and only there is an enhancement in the intensity of I_1_ (the emission intensity at 374 nm), whereas no effect is seen on that of I_3_ (the emission intensity at 395 nm). By formation of micelles in an aqueous solution, pyrene entered to the hydrophobic core of the micelles and the intensity of the third highest vibrational band at 395 nm (I_3_) starts to strongly increase. Therefore, the changes in the intensity ratio of I_1_/I_3_ were used to determine the CMC of stearic acid-depolymerized chitosan micelles. The plot of the intensity ratio of I_1_/I_3_ versus the logarithm of the polymer concentration is exhibited in Figure 4. The interception of the two straight lines was considered as CMC of the polymer and was observed at 1.58×10^-2^ mg/ml of the polymer. This value was similar to the CMC values that were reported in the literature [26,27].

### Entrapment efficiency of ITRA in the polymeric micelle formulations

The amount of drug entrapment in polymeric micelles by physical procedure is depended on several factors, including physicochemical properties of drug and block copolymers [28]. In this study, a long fatty acid chain (stearic acid) was used to entrap itraconazole as a highly hydrophobic molecule.

The polymeric micelles prepared in this study could entrap up to 43.2±2.27 μg/ml of ITRA (Table 1), more than 1000-fold relative to its aqueous solubility. It seemed that in the film hydration method good dispersion of the drug molecules through very thin polymer film helped to efficiently entrap ITRA. The data showed that by increasing in drug percent ratio in both concentrations of polymer the EE% decreased because the initial amount of added drug was high, however the high amount of ITRA encapsulated at both polymer concentration at 50% weight ratio of drug to the polymer.

**Table 1:** **Physicochemical properties of the polymeric micelle formulations prepared by film hydration method** (**mean**±**SD**, **n**=**3**)

	**Polymer concentration**									
**DA**^**a**^	**1 mg**/**ml**					**2 mg**/**ml**				
	**EE%**^**b**^	**Conc.**^**c**^	**DL**^**d**^	**Size(nm)**	**Z**.**P**^**e**^**(mv)**	**EE%**	**Conc.**	**DL**	**Size(nm)**	**Z.P(mv)**
50%	6.45±0.1	32.25±1.6	3.12±0.05	154.25±1.1	60.25±1.9	4.32±0.2	43.2±2.3	4.13±0.21	189.65±1.3	34.2±1.6
16.6%	9.94±0.6	16.51±1.1	1.62±0.10	125.75±1.7	63.25±1.7	6.03±0.4	20.03±1.4	0.99±0.07	176.71±2.6	45.31±1.8
5%	50.66±3.9	25.33±1.9	2.47±0.18	146.95±1.9	71.62±1.1	37.16±2.9	37.16±2.9	1.82±0.14	196.51±1.3	52.95±1.1
Blank micelle	-	-	-	121.37±1.1	74.28±1.8	-	-	-	168.31±1.2	58.34±1.7

^a^ Drug amount per mg of polymer weight, ^b^ Entrapment efficiency, ^c^ Drug concentration in formulation(μg/ml), ^d^ Drug loading, ^e^ Zeta potential.

The highest entrapment efficiency obtained by this procedure (37.16±2.91) when the polymer concentration was high (2 mg/ml) and the drug was used at the lowest percentage.

### Particle size distribution measurements

The molecular weight of polymer and the properties of hydrophobic segment and drug are the most important factors influencing the size of polymeric micelles [29]. In all formulations, the mean diameters of the ITRA loaded polymeric micelles were between 120 to 200 nm with polydispersity index ranged between 0.18-0.22 (Table 1). The size of micelles increased significantly with increasing the polymer concentration. Data showed that significant change has been made with increasing ITRA to polymer weight percent from 5% to 50%, while keeping the polymer concentration constant. The more ITRA entrapped into the blank micelles, the more zeta potential was decreased (Table 1). Generally zeta potential exhibited reverse relation with the amount of ITRA entrapped into the micelles.

### Nebulization of ITRA loaded polymeric micelles

For evaluation of nebulization efficacy, ITRA loaded polymeric micelle formulations were aerosolized at 28 l/min through a twin stage impinger. FPF (ratio of aerosolized particles < 6.4 μm) and the value of nebulization efficiency of the formulations are exhibited in Table 2. The results showed that polymeric micelles with different particle size had the same aerosolization properties.

**Table 2:** **Aerosolization parameters of ITRA loaded polymeric micelles at 2 mg**/**ml polymer concentration** (**mean**±**SD**, **n**=**3**)

**DA**^**a**^	**RD**^**b**^**(%)**	**ED(μg)**	**FPD(μg)**	**FPF (%)**	**DRE(%)**	**NE(%)**
50%	84.05±3.23	197.5±3.53	94.2±3.64	47.7±0.98	89.4±1.5	89.9±0.99
16.6%	80.15±1.20	92.25±4.59	39.7±3.20	43.0±1.32	95.3±1.1	88.9±0.11
5%	84.9±1.97	171±7.07	76.1±1.55	44.5±2.73	98.0±0.6	89.5±0.27

^a^Drug amount per mg of polymer weight, ^b^Recovered Dose.

These results are in accordance with our previous work on amphotericin B polymeric micelle formulations [20]. Also some reports showed that physical properties of the nebulization medium have an important role in the size distribution of the aerosolized droplets and droplets aerosolized from medium with similar properties showed the same size distribution [30]. To calculate the resistance of polymeric micelle formulations against shear force that occur in the air jet nebulizer and to evaluate the capability of these formulation to remained ITRA encapsulated during nebulization, the encapsulation efficiency of the formulations after nebulization were studied. Our results showed that almost all of the formulations were stable during nebulization process (Table 2). These data suggested that polymeric micelles have good stability against shear force induced in air jet nebulizer. Similar results were observed after nebulization of amphotericin B loaded polymeric micelles [20].

### In vitro release study

**Figure 5:**
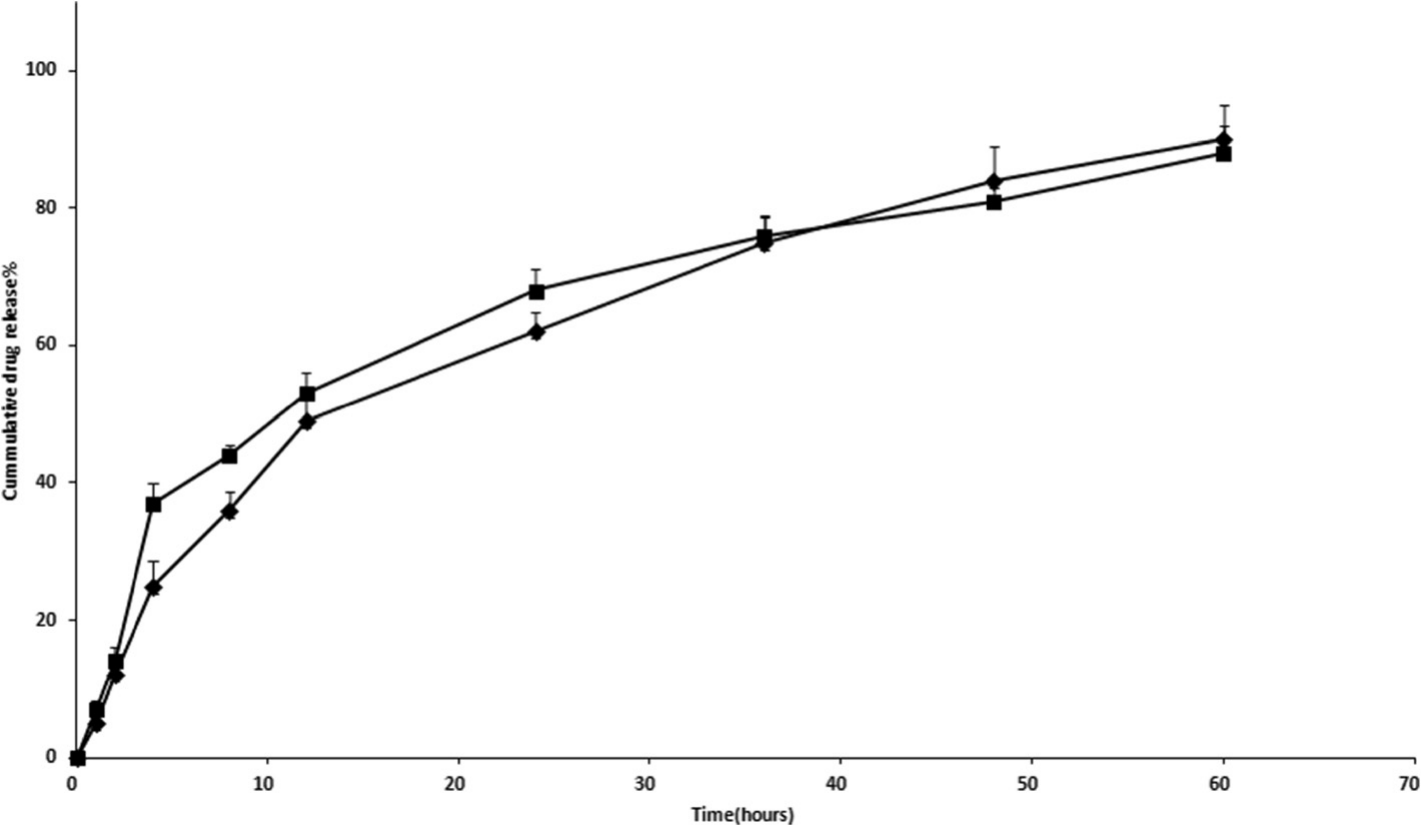
**In vitro ITRA release profile from micelles prepared by different polymer concentration** (♦) **2mg**/**ml and(■)1mg**/**ml with same drug percent ratio(5%).**

In vitro release profile of ITRA from formulation containing 5% drug amount per mg of polymer weight at 1 and 2 mg/ml polymer concentration exhibited in Figure 5. The release profiles showed two phase pattern. Over the first 12 hrs about 49% of the drug released from both micellar formulations. This relative rapid release behavior may be due to the drug molecules which adsorb on the outer surface of the micelles or intercalate between hydrophilic polymers. Total amount of the drug released after 60 hrs. Similar release pattern from polymeric micelles reported in some other studies [31,32]. As it shown in Figure 5 and results obtained from statistical analysis there is no significant difference between release profile of formulations.

### In vitro antifungal activity

Table 3 showed the MIC of ITRA in different formulations for 3 fungal strains. The results showed that ITRA loaded polymeric micelle formulations had the same MIC values as dissolved ITRA in DMSO. The antifungal activity of the two micellar formulations was similar probably due to close proximity of the size distribution data of the formulations. The data obtained from antifungal experiments revealed that chitosan based polymeric micelles could be used for solubilization of ITRA in aquouse media with antifungal activities equal to that of organic based ITRA solutions. The micelles without drug had no significant antifungal activity.

**Table 3:** **Minimal inhibitory concentration of free ITRA** (**dissolved in DMSO**) **and ITRA loaded in polymeric micelles against three fungal strains** (**mean**±**SD**, **n**=**3**)

**ITRA in**	**C**. **albicans ATCC 10231**	**A**. **fumegatus ATCC 13073**	**A**. **niger ATCC 16404**
Micelles 5%*	0.5	2	1
Micelles 50%**	0.5	2	1
DMSO	0.5	2	1

*, Micelles prepared by using 5% initial drug amount per mg of polymer weight at 2 mg/ml initial polymer concentration.

**, Micelles prepared by using 50% initial drug amount per mg of polymer weight at 2 mg/ml initial polymer concentration.

## Conclusion

Through grafting stearic acid onto depolymerized chitosan, the modified polymer can form micelles in nano size range. The data showed that stearic acid as the hydrophobic core of the micelles could entrap ITRA and increase the solubility of the drug. The size distribution of all formulations was mono modal and ranged from 120 nm to 200 nm. In vitro nebulization study of the ITRA loaded formulations showed that the stearic acid-chitosan based polymeric micelles had adequate capability as nanocarriers to deliver ITRA and can remain their stability during nebulization.

## Competing interests

The authors report no conflicts of interest. The authors alone are responsible for the content and writing of the paper.

## Authors’ contributions

EM: Carried out synthesis studies of polymer, micelle preparation, different characterization of micelles and drafted the manuscript. KM: Supervisor and participated in the drafted the manuscript. ARN, MRR: Supervisor. NM: Carried out in vitro pulmonary studies. MA: Supervisor and participated in polymer characterization. MAB: Carried out characterization of synthesized polymer. All authors read and approved the final manuscript.
